# Supplementary material for: Integrating climate change induced flood risk into future population projections
Source: Nat Commun. 2023 Dec 18;14:7870. doi: 10.1038/s41467-023-43493-8 (PMC10728110; doi:10.1038/s41467-023-43493-8)
Supplement: Supplementary file 1 — Supplementary Information [file 41467_2023_43493_MOESM1_ESM.pdf]

Supplementary Information

Data and Methods

Supplementary Table 1. Data Sources

Data Class	Resolution	Time Period	Source
Flood Exposure: inundation within the 5, 20, 100, and 500-year RPs.	Block	2023, 2053	First Street Foundation - aggregated property inundation from FSF Flood Model[1]
Historic Flood Exposure: counts of historic events obtained from NLP of media coverage of flood events	Tract	2000-2020	Lai et al.[2] and NOAA Storm Events Database[3]
Baseline population projections for every 5 year interval from 2020 to 2100 under all SSPs	County (proportionally downscaled to block)	2020-2100	Hauer, 2019[4] available at doi.org/10.17605/OSF.IO/9YNFC
Population counts	Block	2000, 2010, 2020	Census decennial
Multidimensional Deprivation Index	County	2010-2019	Glassman[5]
Job Growth Rate	Tract	2004-2013	Average annualized growth rate from Longitudinal Employer-Household Dynamics and Local Area Unemployment Statistics (compiled by Chetty et al.[6])
Job density	Tract	2013	Number of jobs per square mile in this area in 2013 from 2010 Census Gazetteer, Longitudinal Employer-Household Dynamics and Local Area Unemployment Statistics (compiled by Chetty et al.[6])
Population density	Tract	2010	Residents per square mile in this area in 2010 from 2010 Census Gazetteer and 2010 Decennial Census (compiled by Chetty et al.[6])
Median income	Tract	2018	ACS 2018

Median home valuation	Tract	2018	ACS 2018
Labor force (employed, unemployed)	Tract	2018	ACS 2018
Proximity to rivers	Spatial, used distance matrix to centroids of tracts	2013	UNC Rivers Dataset[7] available at doi.org/10.1002/wrcr.20440
Proximity to coasts	Spatial, used distance matrix to centroids of tracts	2013	NOAA Coastline File[8] available at doi:10.1029/96JB00104.

Propensity Matching (Step 1 Supplemental Information)

The variables for the propensity score themselves are informed using several sources. Importantly, propensity score modeling is commonly used and most effective when it accurately predicts the treatment. As we are defining our treatment as the block exposure to some influential level of flooding, the most obvious variables to include for this score reflect amenities provided by the proximity to water sources as well as any other characteristics associated with this water proximity. We include distance to the nearest coastline[8] and the proximity to the nearest river source for large rivers, defined by discharge and flow parameters[7]. Additional variables which may influence people living in areas with a certain amount of flooding include certain social and structural amenities[9], which are captured by variables such as a county's MDI (Multidimensional Deprivation Index[5]), density of job opportunities[6] (proxy which captures job market and urbanization characteristics), the population of a block (proxy from 2000 as a gravity indicator, where people are attracted to areas with many other people around), population density in 2010 (proxy for urbanization and social opportunities/amenities), the median income for a tract where some people cannot afford to live in nearby areas without meaningful flood exposure (ACS 2018), median home value where people vary in their willingness to rebuild homes after flood damage (ACS 2018), job growth rates between 2004 and 2013[6] (proxy for urbanization and opportunity), and employment rates (ACS 2018). Our identifying assumption is that conditional on the balance of these observable characteristics, the probability that a block experiences some influential level of flooding is random.

Historic Modeling (Step 2 Supplemental Information)

There were multiple dependent variables considered to represent change in populations, but ultimately block change was selected as it could be most reliably applied to our baseline future projections by using a varying count of years. In this calculation, the block change metric is the result of the difference in time 1 and time 2 population (2000 and 2020 in this case) divided by the population at time 1.

Model Assessment and Validation

To evaluate the prediction methodology, the same methodology described above is used but trained only on a smaller sample set of the data from 2000 to 2020. The data are split into training and testing sets through a stratification process considering their amounts of inundated properties during different flood RPs, job densities, job growth rates, population densities, median incomes, median home values, employment rates, MDI scores, distance to rivers, distance to coasts, future change in the baseline scenario, and the dependent variable of block change. This is done separately for each state, where 80% of the total sample is used to construct the training sample, and the remaining 20% serves as the testing sample set. Using the same approach as detailed above, the appropriate variables to include in the model are selected individually for each state (as determined by the model which has the lowest AIC for each). The fitted model for the training set is then used to create predictions for the testing set by which the observed results are compared against the predictions. During this process, multiple rebalancing approaches were tested to ensure that overall population counts resulting from the model modification matched those from the baseline expectations (here, the historical true counts for an overall area). The rebalancing approaches tested included using county, Core Based Statistical Area (CBSA), and state counts as baselines for redistributing population forecasts that were predicted to be too high, or too low, due to the application of scoring block level population counts from the statistical model coefficient estimates.

The Mean Average Error (MAE), Mean Squared Error (MSE), and R squared are calculated at the county level for block projection observations. Supplementary table 2 and supplementary table 3 report the spread of the county MAE and MSE values for the country (respectively). For the median county, the MAE values are fairly low for all three rebalancing approaches. Generally, the MAE indicated the best performance when rebalancing was done using the county population counts or CBSA population counts. When rebalancing is done at the county level, a median county has an MAE of 0.00 for population counts, and a mean of 0.83. The MAE for block growth rates for a median county (when rebalancing at the county level) has a median of 0.00 and mean of 1.18. This approach is supported by findings that individuals tend to live close to where they grew up, where 80% of young adults live within 100 miles and 90% of individuals live within 500 miles of their hometowns[10]. When rebalancing is done at the CBSA level, a median county has an MAE of 0.09 for population counts, and a mean of 0.80. The MAE for block growth rates for a median county (when rebalancing at the CBSA level) has a median of 0.04 and mean of 0.90. You can see similar patterns when evaluating the MSE and R-Square metrics.

Supplementary Table 2. Country MAE spread for blocks within counties. There are 4 NA values when rebalancing was done by County. There are 1180 NA values when rebalancing was done by CBSA.

		Min.	1st Qu.	Median	Mean	3rd Qu.	Max.
**MAE for block**	**Rebalancing by County***	0.00	0.00	0.00	0.83	1.58	11.99

**counts**	**Rebalancing by CBSA****	0.00	0.00	0.09	0.80	1.39	13.26
	**Rebalancing by State**	0.00	0.42	1.16	1.62	1.88	97.29
**MAE for block growth rates**	**Rebalancing by County***	0.00	0.00	0.00	1.18	2.27	13.34
	**Rebalancing by CBSA****	0.00	0.00	0.04	0.90	1.53	14.98
	**Rebalancing by State**	0.00	0.33	1.02	1.51	2.34	24.45

Supplementary Table 3. Country MSE spread for blocks within counties. There are 4 NA values when rebalancing was done by County. There are 1180 NA values when rebalancing was done by CBSA.

		**Min.**	**1st Qu.**	**Median**	**Mean**	**3rd Qu.**	**Max.**
**MSE for block counts**	**Rebalancing by County***	0.00	0.00	0.00	3.12	4.38	210.45
	**Rebalancing by CBSA****	0.00	0.00	0.01	4.01	4.05	204.86
	**Rebalancing by State**	0.00	0.27	2.78	20.61	7.13	9466.18
**MSE for block growth rates**	**Rebalancing by County***	0.00	0.00	0.00	7.81	9.92	426.54
	**Rebalancing by CBSA****	0.00	0.00	0.00	5.59	4.73	224.28
	**Rebalancing by State**	0.00	0.12	1.77	10.35	11.79	598.00

Supplementary figure 1 further investigates the spatial distribution of the error captured in the MAE and MSE metrics above. The results indicate that there is a relatively low amount of error across the country and that the spatial correlation of that error is relatively low. In particular, the images highlight a disproportionately high level of shading in the lowest MAE and MSE categories (approaching 0) across the entire country. There is a slight concern over the spatial correlation of the error, which tends to indicate that there is likely some omitted variable issues with the models as they have been specified to this point. However, there is not a clear spatial pattern and the relative levels of the reported Moran's I values (testing for spatial dependence) are low enough to indicate a relatively weak correlation with the omitted variables in the analysis.

Supplementary Figure 1. Spatial Distribution of MAE and MSE for Population Growth and Count. Data were tested for spatial autocorrelation and there are very weak levels as evidenced by the Moran's I statistics ranging from 0.02 - 0.27. This indicates that there is some spatial clustering around the goodness of model fit, but that the spatial variation is weak (in absolute terms) and its impact on model fidelity is negligible. Spatial layers were obtained from the U.S. Census Bureau.

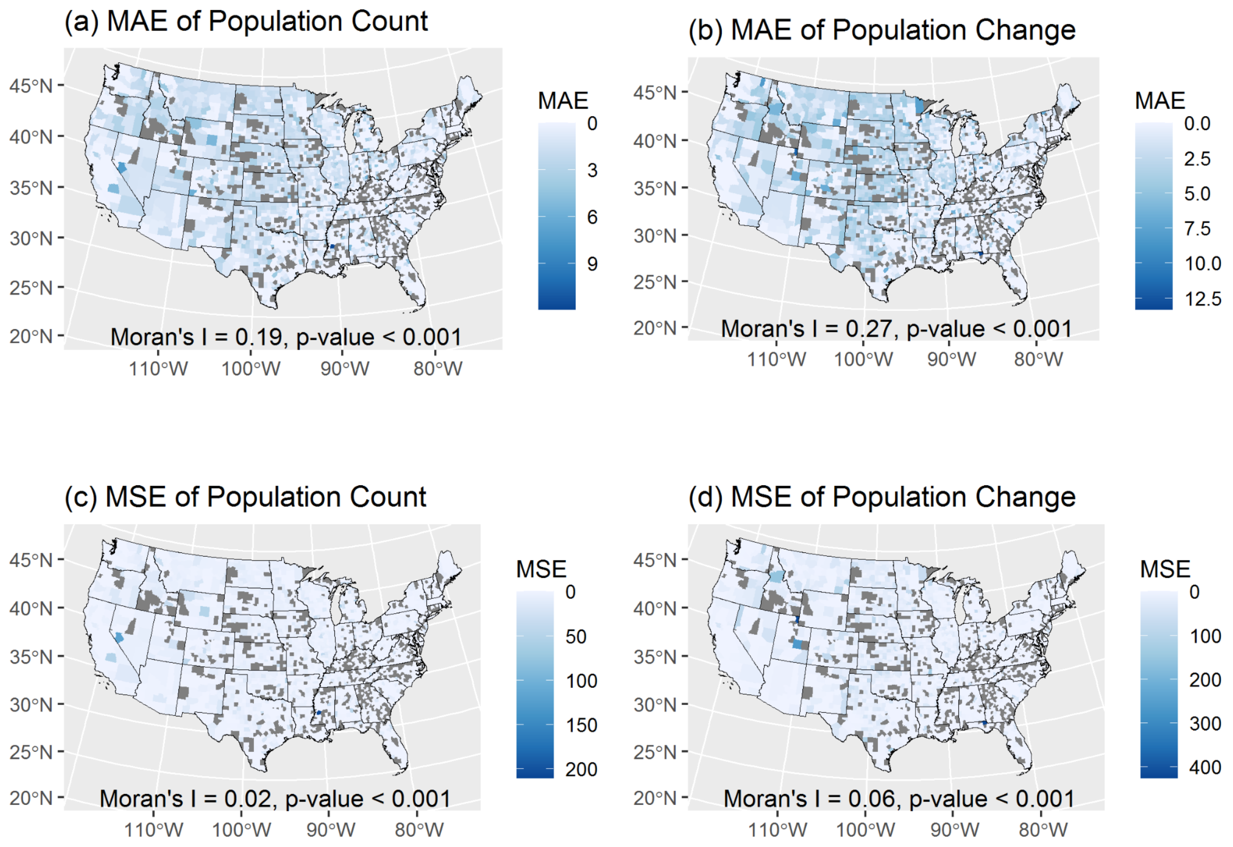

Theoretically, population redistribution patterns have generally been driven by the push-pull factors of places (both cities and rural areas alike) and the character of those places. Supplementary figure 2 further investigates any proclivity of the model to over-emphasize the characteristics of different population sizes. The results indicate that the MAE and MSE levels are relatively evenly distributed across counties when grouped into quartiles by population size. In fact, there is almost no difference across the metric being modeled (population count vs. population change) based purely on the structure of the error distributions in the figure, moving from left to right. However, the MSE metrics do seem to indicate that there are a few outliers that are decompressing the IQR distribution. Overall, the statistical distribution seems to align well with the spatial distribution in that there seems to be evidence that the models used to understand the historical relationship between flood exposure are good predictors of actual known population change metrics over that same time period.

Supplementary Figure 2. Statistical Distribution of MAE and MSE for population estimates. Panels show MAE and MSE spread for population growth rates (chg) and population count (n). Results are shown for population quartiles.

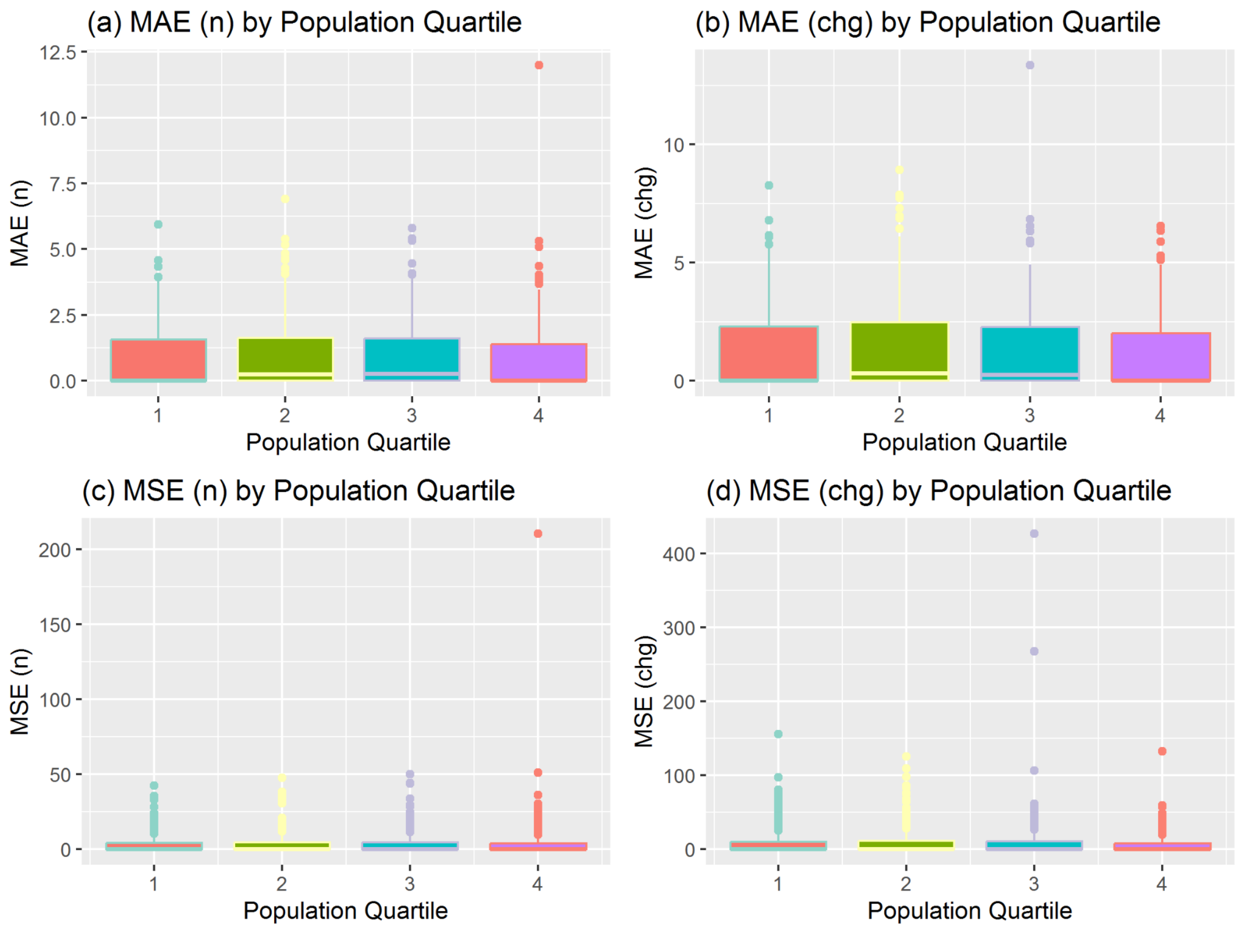

Given the evaluation of MAE, MSE, and R-squared, as well as the conclusions that they are all absolutely low and don't seem to have an systematic patterns, the coefficient estimates were deemed to reliably useful in the application of forecasting future population levels given the levels of changing flood exposure forecasted by the FSF-FM.

Future Projections (Step 3 Supplemental Information)

The future baseline population projections inherently hold the assumptions of attractiveness for various cohorts of populations (such as family attractiveness) and implicitly capture amenity provision and other growth factors assuming relatively no changes to local environmental conditions or other factors of similar influential scales. That is, the projections are based on Cohort-Change-Ratios (CCRs) and Cohort-Change-Differences (CCDs), and capped at the population counts projected for various SSPs[4]. The population counts that align with the SSP

population count projections (at which the baseline numbers are capped at) are provided at the country-wide level, which capture the country's mitigation abilities, adaptation abilities, and population exposure to hazards relative to other countries[11].

Supplemental Results

Supplementary figure 3 illustrates the relationship between the same three different flood exposure indicators (5, 20, and all RPs) and change in population against the 2020 starting point at the state level. While there are underlying variations across the state (presented below), this indicates that there seems to be a relatively clear divide between coastal and inland states in regard to future population growth. Most notably, the treatment groups grow at a slower absolute level in the states of California, Texas, and Florida. In these high population states, we'd expect population growth across the board but the results find that larger growth across the state is expected to occur in areas with less vulnerability to flooding, across all three displayed binary indicators. On the other hand, there are some states like Ohio and Maryland where the results of this study show that all of the aggregate growth is expected to occur in areas where the proportion of inundated properties for the three different indicator options is higher than the average across the state. In particular, Ohio actually shows a decrease in population in areas which are less exposed (through the three indicators) and an increase in areas that have high exposure. There are a series of reasons that we see these types of effects at the aggregated state level. First, is that there is a large amount of spatial variability *within* the state and in places with relatively low flood exposure, or exposure that is primarily inland, there is less awareness and preparation for the actual exposure. Additionally, much of the riverine and rainfall exposure in these particular inland regions tends to be very concentrated and at a less frequent level than the 5 and 20-year RPs, for both the current year and under future climate projections, which drive the largest responses in regards to community level population change. Recent research has highlighted that inland flood risk/exposure is increasing[12]. This increase is uneven around the country, but the Midwest, Northeast, and Texas/Louisiana Gulf Coast are likely to be the most impacted by increasing inland flooding driven primarily by the increased likelihood and severity of extreme precipitation events. As such, we expect inland areas to increasingly respond to the higher frequency of flood exposure into the future. Finally, states like North Carolina with exceptional exposure in areas like the barrier islands are already starting to see retreat bear out in the historic models, which is further exacerbated by sea-level rise and the increasingly latitudinal reach of cyclonic activity.

Supplementary Figure 3. Absolute difference between future projections and current populations by treatment class. Panels show these differences by different dichotomous group breaks based on the following definitions: if a block has higher than average proportion of inundated properties in the (a) 5, 20, or 100-year RPs, (b) 20-year RP, and (c) 5-year RP.

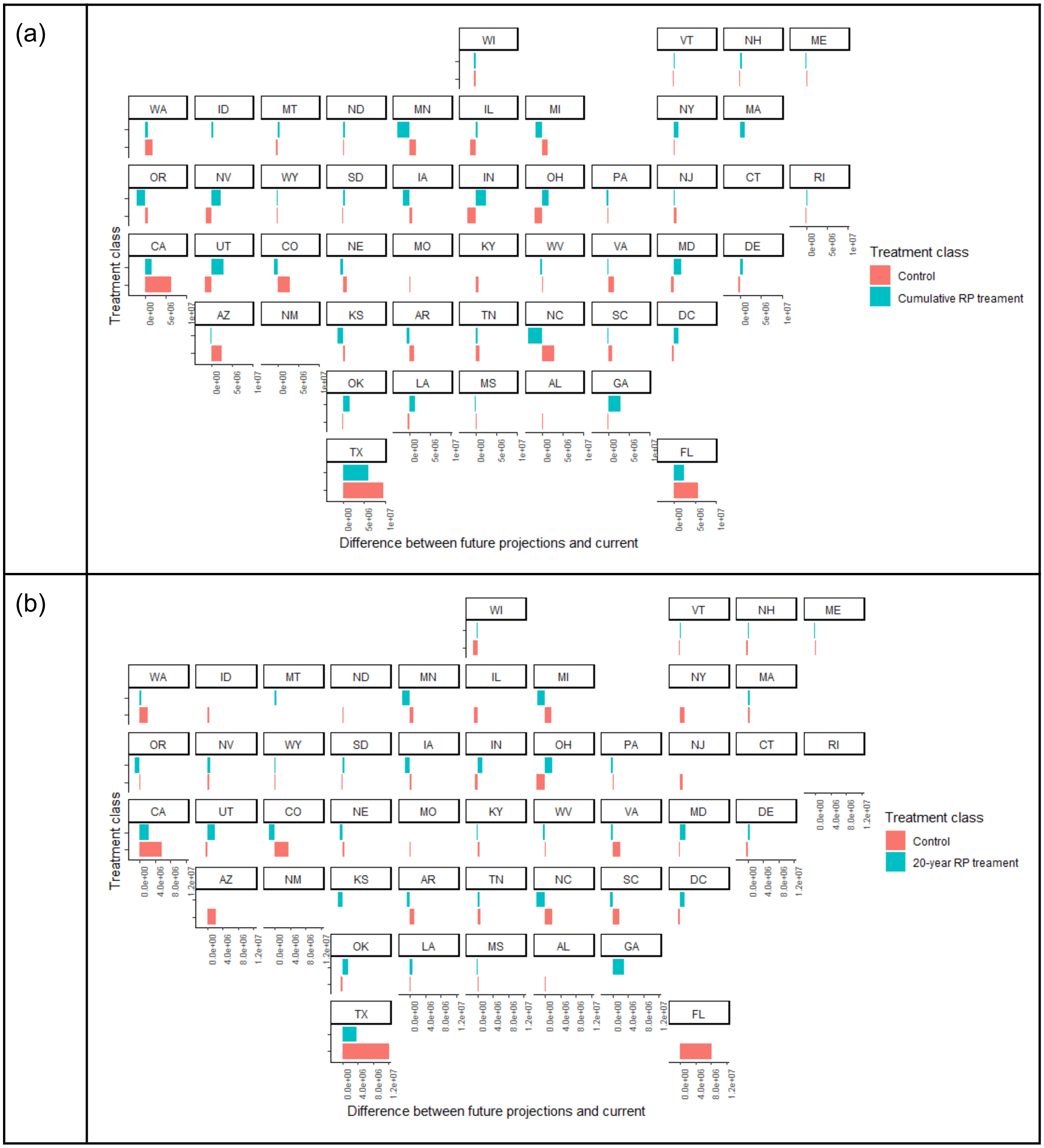

(c)

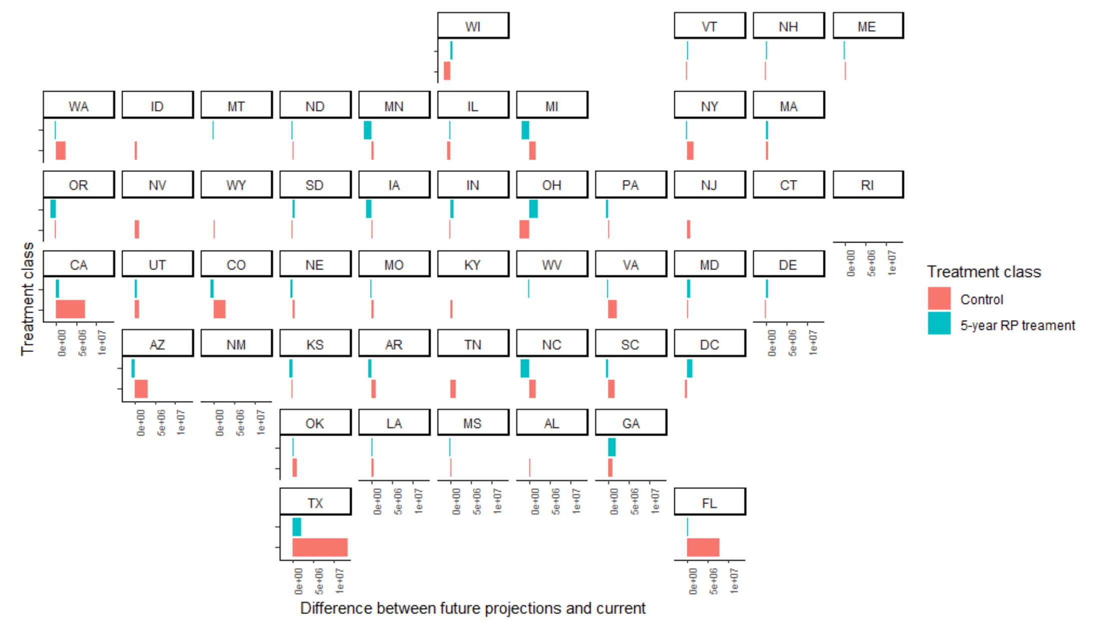

Supplementary figure 4 further illustrates the projected absolute population levels compared to baseline projections by state (projected - baseline). The figure shows that there are decreases in the projections compared to baseline in the counts of the population living in highly vulnerable areas in California, Florida, Colorado, South Carolina, and North Carolina. Again these numbers hide the underlying variation from the smaller groups used to produce these aggregates, but when taken in conjunction with the panels from Supplementary figure 3 above they tell a story of an overall slower growing, or even decreased, population in areas with relatively high exposure to flooding. Based on the integration of the historic relationships in these areas between flood exposure and population change responses, this figure further reports that these vulnerable areas generally experience a negative climate consequence on their population growth, when compared to a generally positive climate consequence effect in areas that are not as exposed to flooding. Additionally, variations in the exposure profiles of states in regards to the frequency with which they are exposed to flood events will also directly impact the differential results that are reported here. The following sets of results delve into those differences by exposure profile and levels of spatial aggregation.

Supplementary Figure 4. Difference between future projections and baseline projections by treatment class. Panels show these differences by different dichotomous group breaks based on the following definitions: if a block has higher than average proportion of inundated properties in the (a) 5, 20, or 100-year RPs, (b) 20-year RP, and (c) 5-year RP.

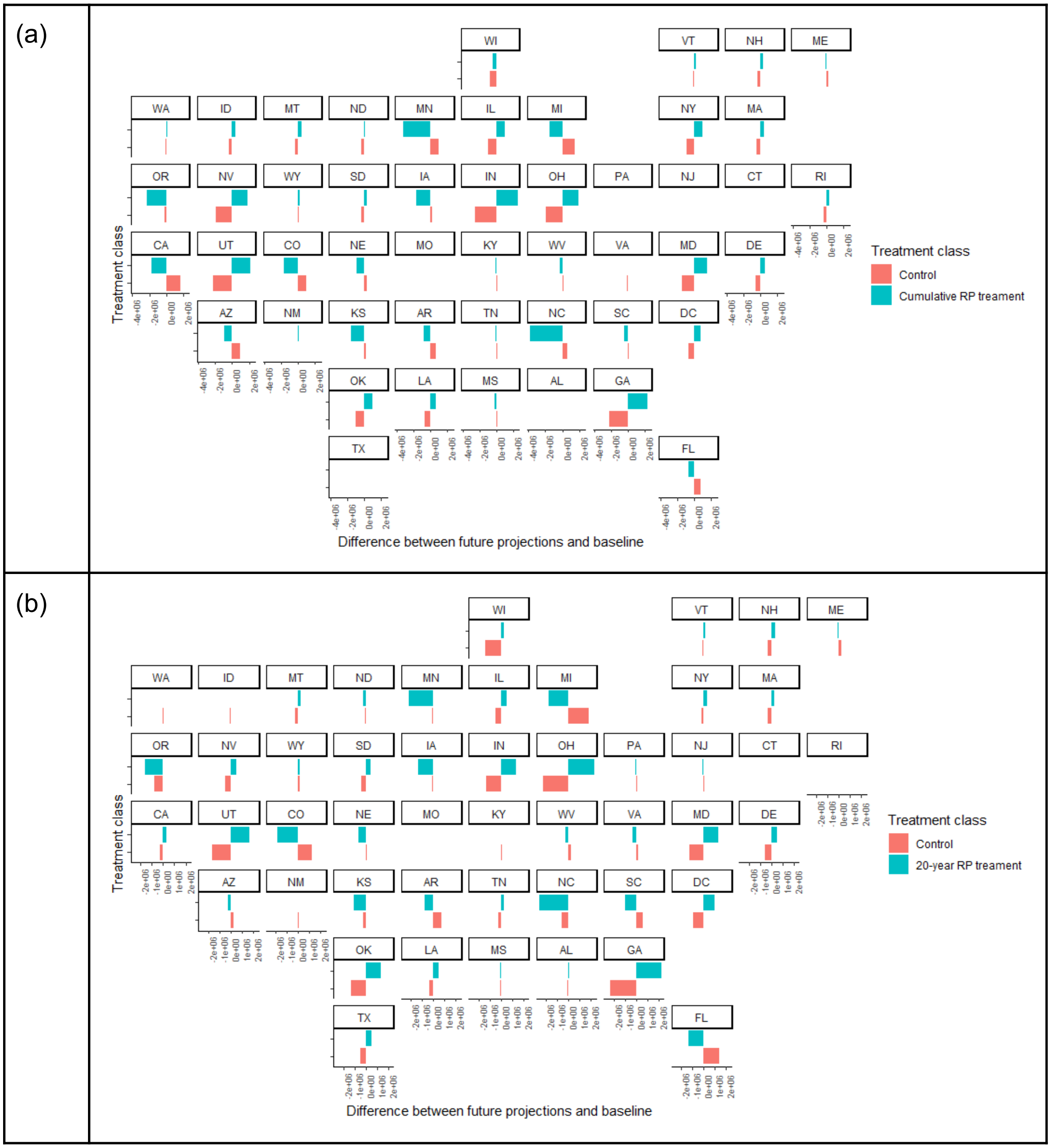

(c)

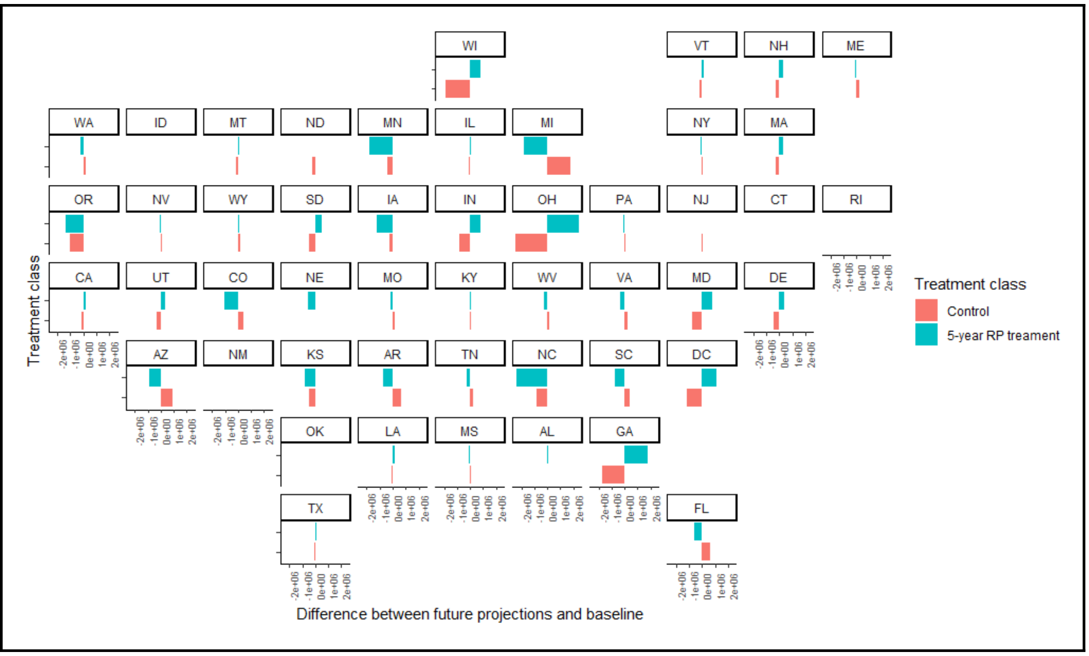

The results indicate that there is within state variability in regards to the areas that are forecast to gain or lose populations into the future. Of particular interest is that locations in south FL and TX's Gulf Coast are forecast to continue to see population growth on average. Other locations, like the Mid-Atlantic regions of southern NJ, southeastern NY, eastern MD, and coastal NC are forecast to see population declines in their most flood prone areas.

Results for Cincinnati, OH, are presented here to illustrate the integration of the modeled climate consequence into future population projections for inland flooding. Supplementary figure 5 highlights that an opposite pattern exists in the Cincinnati example. Much of the muted pattern in Cincinnati is related to the relatively small changes in flood risk across the 30 year time period. This is indicative of some of the larger national patterns which indicate that coastal areas, especially along the Gulf and Southeast Atlantic coast are likely to see the largest increase in flood risk over this time period, while inland areas will see more modest increases in flood risk. That being said, the figure does show a non-linear pattern associated with changing risk and population shifts. In the 20-year RP there is a small bump in population change up to about a 1% increase in flood risk, at which point there is a very shallow decline in population. This pattern is relatively similar in regard to the 100-year RP where there is a sharper increase in populations up to about 1% and then a steeper negative effect. The pattern is most pronounced in the 100-year exposure for all the reasons related to frequency and severity above.

Supplementary Figure 5. Relationship between exposure within the (a) 20 and (b) 100-year RP with population change, future forecasted population change between 2023 - 2053 (Cincinnati, OH). Data are presented as mean values with 95% confidence, with a randomized sample of future flood exposure and population counts.

(a) (b)

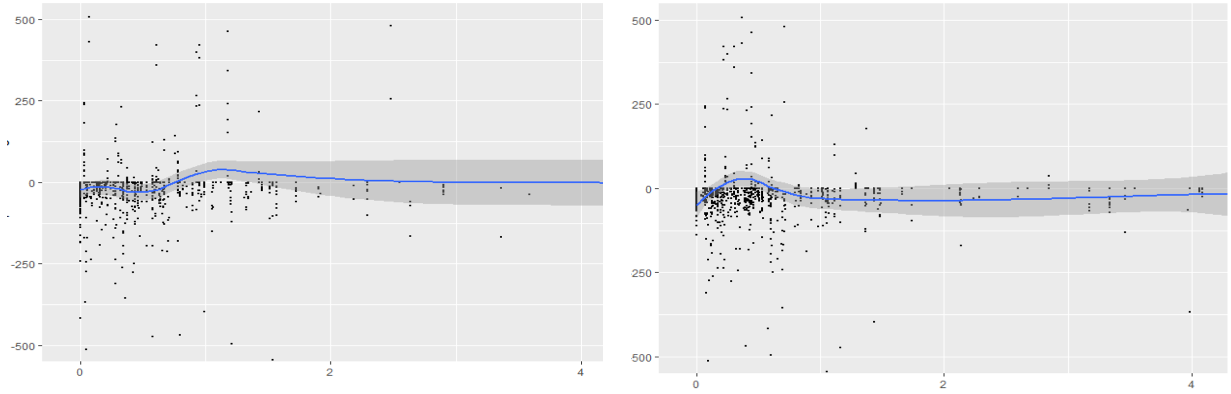

Supplementary figure 6 highlights the relationship between forecast population change and changing flood exposure (by selected RP scenarios) out to mid-century. While in Miami-Dade, there is projected to be growth that is negatively correlated with the exposure identified in the 100-year flood zone, an opposite pattern exists in Cincinnati, where the patterns of population growth seem to follow areas of particularly high exposure in the 100-year flood zone. This highlights that when seeking to understand the future impact of flood exposure change, using high precision data in regard to both flood exposure and population change, are required to see an adequate picture of the impacts. The areas along the Ohio River in Cincinnati, OH are those most susceptible to increased flooding and actually see population growth projected out to 2053. Again, this is primarily due to the nature of inland flooding as both less frequent and less severe in combination relative to the coastal area.

Supplementary Figure 6. County level projected population change resulting from the application of the climate consequence to the SSP2 future population projections (Cincinnati, OH). Census Block Projections of: (a) Population Change with the addition of modeled historic relationships between population and flood exposure, and (b) Flood Exposure of Properties in the 100-year Flood Zone. Base Maps are provided by OpenMapTiles, CARTO, and OpenStreetMap contributors. Spatial block layers were obtained from the U.S. Census Bureau.

(a)

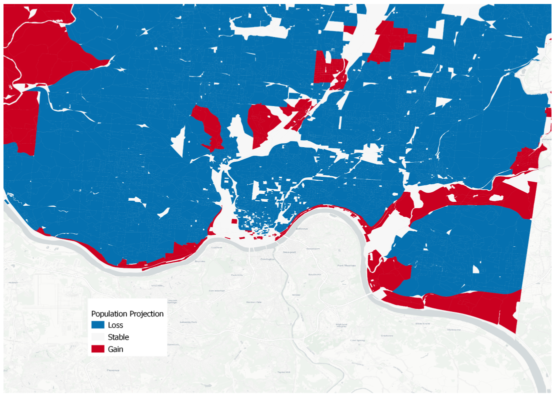

(b)

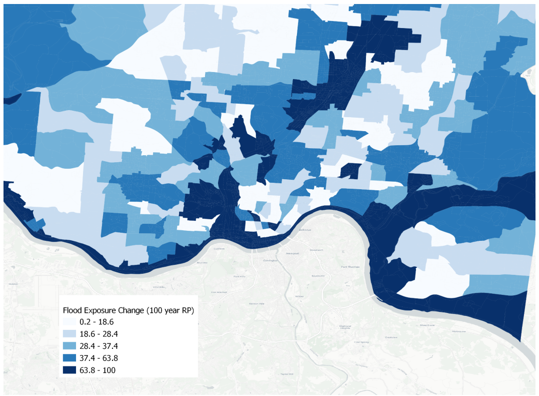

Supplementary Information References:

[1] First Street Foundation. First Street Foundation Technical Documentation. 2020. Available online: https://assets.firststreet.org/uploads/2020/06/FSF_Flood_Model_Technical_Documentation.pdf (accessed on 28 June 2023).

[2] Lai, K., Porter, J. R., Amodeo, M., Miller, D., Marston, M., & Armal, S. (2022). A natural language processing approach to understanding context in the extraction and geocoding of historical floods, storms, and adaptation measures. *Information Processing & Management*, *59*(1), 102735.

[3] NOAA Storm Events Database (accessed in January 2023). https://www.ncdc.noaa.gov/stormevents/ftp.jsp

[4] Hauer, M. E. (2019). Population projections for US counties by age, sex, and race controlled to shared socioeconomic pathway. *Scientific data*, *6*(1), 1-15.

[5] Glassman, B. (2020). The multidimensional deprivation index using different neighborhood quality definitions. *United States Census Bureau Social, Economic, and Housing Statistics Division*.

[6] Chetty, R., Friedman, J. N., Hendren, N., Jones, M. R., & Porter, S. R. (2018). *The opportunity atlas: Mapping the childhood roots of social mobility* (No. w25147). National Bureau of Economic Research.

[7] Andreadis, K. M., Schumann, G. J. P., & Pavelsky, T. (2013). A simple global river bankfull width and depth database. *Water Resources Research*, *49*(10), 7164-7168.

[8] Wessel, P., and W. H. F. Smith (1996), A global, self-consistent, hierarchical, high-resolution shoreline database, J. Geophys. Res., 101(B4), 8741–8743, doi:10.1029/96JB00104.

[9] Waltert, F., & Schläpfer, F. (2010). Landscape amenities and local development: A review of migration, regional economic and hedonic pricing studies. *Ecological Economics*, *70*(2), 141-152.

[10] Sprung-Keyser, B., Hendren, N., & Porter, S. (2022). *The Radius of Economic Opportunity: Evidence from Migration and Local Labor Markets* (No. 22-27).

[11] Samir, K. C., & Lutz, W. (2017). The human core of the shared socioeconomic pathways: Population scenarios by age, sex and level of education for all countries to 2100. *Global Environmental Change*, *42*, 181-192.

[12] Kim, J., Shu, E., Lai, K., Amodeo, M., Porter, J., & Kearns, E. (2022). Assessment of the standard precipitation frequency estimates in the United States. *Journal of Hydrology: Regional Studies*, *44*, 101276.
