# Supplementary material for: Integrating climate change induced flood risk into future population projections
Source: Nat Commun. 2023 Dec 18;14:7870. doi: 10.1038/s41467-023-43493-8 (PMC10728110; doi:10.1038/s41467-023-43493-8)
Supplement: Supplementary file 3 — Reporting Summary [file 41467_2023_43493_MOESM3_ESM.pdf]

nature portfolio

Corresponding author(s): Evelyn G. Shu

Last updated by author(s): 2023 - 10 - 30

Reporting Summary

Nature Portfolio wishes to improve the reproducibility of the work that we publish. This form provides structure for consistency and transparency in reporting. For further information on Nature Portfolio policies, see our Editorial Policies and the Editorial Policy Checklist.

Statistics

For all statistical analyses, confirm that the following items are present in the figure legend, table legend, main text, or Methods section.

n/a	Confirmed	
[ ]	[x]	The exact sample size (*n*) for each experimental group/condition, given as a discrete number and unit of measurement
[ ]	[x]	A statement on whether measurements were taken from distinct samples or whether the same sample was measured repeatedly
[x]	[ ]	The statistical test(s) used AND whether they are one- or two-sided<br>*Only common tests should be described solely by name; describe more complex techniques in the Methods section.*
[ ]	[x]	A description of all covariates tested
[ ]	[x]	A description of any assumptions or corrections, such as tests of normality and adjustment for multiple comparisons
[ ]	[x]	A full description of the statistical parameters including central tendency (e.g. means) or other basic estimates (e.g. regression coefficient) AND variation (e.g. standard deviation) or associated estimates of uncertainty (e.g. confidence intervals)
[x]	[ ]	For null hypothesis testing, the test statistic (e.g. *F*, *t*, *r*) with confidence intervals, effect sizes, degrees of freedom and *P* value noted<br>*Give P values as exact values whenever suitable.*
[x]	[ ]	For Bayesian analysis, information on the choice of priors and Markov chain Monte Carlo settings
[ ]	[x]	For hierarchical and complex designs, identification of the appropriate level for tests and full reporting of outcomes
[x]	[ ]	Estimates of effect sizes (e.g. Cohen's *d*, Pearson's *r*), indicating how they were calculated

Our web collection on statistics for biologists contains articles on many of the points above.

Software and code

Policy information about availability of computer code

Data collection	Data for this analysis were collected from multiple sources. Historic flood exposure data were obtained via natural language processing techniques in python and these methods are contained in detail in another paper cited in the manuscript (Lai et. al 2022). Current and future flood risk data were obtained from the First Street Foundation and no software was required in the collection of that data. All covariates included in the regression models were obtained from public government sources, including the US Census Bureau, Economic Research Service, and the Bureau of Economic Analysis and no software was required. Future population projections are publicly available at the county level at the NASA SEDAC website. Finally, all GIS and spatial layers were obtained from the US Census Bureau and no software was required.
Data analysis	All data analyses contained in the manuscript were conducted using R and R-studio version 4.2.

For manuscripts utilizing custom algorithms or software that are central to the research but not yet described in published literature, software must be made available to editors and reviewers. We strongly encourage code deposition in a community repository (e.g. GitHub). See the Nature Portfolio guidelines for submitting code & software for further information.

Data

Policy information about availability of data

All manuscripts must include a data availability statement. This statement should provide the following information, where applicable:

- Accession codes, unique identifiers, or web links for publicly available datasets
- A description of any restrictions on data availability
- For clinical datasets or third party data, please ensure that the statement adheres to our policy

The input datasets used for this analysis are either already publicly available or cannot be made available due to restrictive data sharing agreements. All data sources are listed with links to access points in Appendix Table 1.

Research involving human participants, their data, or biological material

Policy information about studies with human participants or human data. See also policy information about sex, gender (identity/presentation), and sexual orientation and race, ethnicity and racism.

Reporting on sex and gender	n/a - secondary aggregated analysis focused on area population total counts only
Reporting on race, ethnicity, or other socially relevant groupings	n/a - secondary aggregated analysis focused on area population total counts only
Population characteristics	n/a - secondary aggregated analysis focused on area population total counts only
Recruitment	n/a - secondary aggregated analysis focused on area population total counts only
Ethics oversight	n/a - secondary aggregated analysis focused on area population total counts only

Note that full information on the approval of the study protocol must also be provided in the manuscript.

Field-specific reporting

Please select the one below that is the best fit for your research. If you are not sure, read the appropriate sections before making your selection.

☐ Life sciences ☒ Behavioural & social sciences ☐ Ecological, evolutionary & environmental sciences

For a reference copy of the document with all sections, see nature.com/documents/nr-reporting-summary-flat.pdf

Behavioural & social sciences study design

All studies must disclose on these points even when the disclosure is negative.

Study description	Quantitative analysis modeling the historic relationship between flood exposure/risk and population change followed by prospective forecasting of future population based on future risk expectations.
Research sample	The data employed in the analysis represent the full population of US Census Block across the US. The modeling procedures employed a control-treatment modeling approach including all Census Blocks with flood risk and a matching set of control Census Blocks selected through a propensity matching algorithm.
Sampling strategy	The data represent the full set of US Census Blocks in the US. Where sampling was employed, a propensity matching technique was used to create a control-treatment group for the modeling phase of the analysis. The researchers were not blinded to the experimental conditions as they were set based on statistical thresholds and the subsequent propensity match technique.
Data collection	Data for this analysis were collected from multiple sources. Historic flood exposure data were obtained via natural language processing techniques in python and these methods are contained in detail in another paper cited in the manuscript (Lai et. al 2022). Current and future flood risk data were obtained from the First Street Foundation and no software was required in the collection of that data. All covariates included in the regression models were obtained from public government sources, including the US Census Bureau, Economic Research Service, and the Bureau of Economic Analysis and no software was required. Future population projections are publicly available at the county level at the NASA SEDAC website. Finally, all GIS and spatial layers were obtained from the US Census Bureau and no software was required.
Timing	Data were collected between June 2022 and February 2023
Data exclusions	No data were excluded.
Non-participation	n/a - This research uses data for the entire contiguous US.

Randomization	Randomization was not employed in this analysis. Instead, the use of all US Census Blocks across the US were employed and a control-treatment design was employed for the modeling phase in which Block with significant flood risk were matched to blocks with similar characteristics but without flood risk in a control-treatment design.

Reporting for specific materials, systems and methods

We require information from authors about some types of materials, experimental systems and methods used in many studies. Here, indicate whether each material, system or method listed is relevant to your study. If you are not sure if a list item applies to your research, read the appropriate section before selecting a response.

Materials & experimental systems

n/a	Involved in the study
☒	☐ Antibodies
☒	☐ Eukaryotic cell lines
☒	☐ Palaeontology and archaeology
☒	☐ Animals and other organisms
☒	☐ Clinical data
☒	☐ Dual use research of concern
☒	☐ Plants

Methods

n/a	Involved in the study
☒	☐ ChIP-seq
☒	☐ Flow cytometry
☒	☐ MRI-based neuroimaging

Plants

Seed stocks	*Report on the source of all seed stocks or other plant material used. If applicable, state the seed stock centre and catalogue number. If plant specimens were collected from the field, describe the collection location, date and sampling procedures.*
Novel plant genotypes	*Describe the methods by which all novel plant genotypes were produced. This includes those generated by transgenic approaches, gene editing, chemical/radiation-based mutagenesis and hybridization. For transgenic lines, describe the transformation method, the number of independent lines analyzed and the generation upon which experiments were performed. For gene-edited lines, describe the editor used, the endogenous sequence targeted for editing, the targeting guide RNA sequence (if applicable) and how the editor was applied.*
Authentication	*Describe any authentication procedures for each seed stock used or novel genotype generated. Describe any experiments used to assess the effect of a mutation and, where applicable, how potential secondary effects (e.g. second site T-DNA insertions, mosiacism, off-target gene editing) were examined.*
